# Cisplatin enhances radiation-induced apoptosis in peripheral lymphocytes of cancer patients: in vivo and in vitro findings

**DOI:** 10.1186/s13014-026-02921-x

**Published:** 2026-09-05

**Authors:** Ali Sak, Emil Mladenov, Yasemin Alberti, Martin Stuschke

**Affiliations:** Department of Radiotherapy, University Hospital Essen, 45122 Essen, Germany

**Keywords:** Lymphocytes, Radiotherapy, Cisplatin, Apoptosis, H2AX, NSCLC

## Abstract

**Background:**

Concurrent cisplatin and radiotherapy frequently cause severe acute hematologic toxicity. This study investigated whether lymphocyte apoptosis, quantified via the γ-H2AX pan-nuclear immunostaining assay, can serve as a reliable biomarker for the acute hematologic side effects of combined chemoradiation.

**Methods:**

Blood samples were collected from cancer patients and healthy controls. Lymphocyte apoptosis was evaluated using the γ-H2AX pan-nuclear assay following in vitro and in vivo exposure to cisplatin and ionizing radiation (IR). In PBMCs, γ-H2AX results strongly correlated with conventional caspase-3 (*r* = 0.9736) and annexin V (*r* = 0.9780) apoptosis assays. Additionally, in vitro experiments assessed how phytohemagglutinin-L (PHA-L)-induced lymphocyte proliferation influences the combined effects of cisplatin and IR on apoptosis, alongside the regulatory role of the ATM protein.

**Results:**

In vitro, cisplatin increased lymphocyte apoptosis asymptotically, plateauing at 10 µg/ml (11.6 ± 5.0%). Combined cisplatin and IR treatment increased apoptosis in a strictly additive manner (radiation effect: *p* < 0.0001), with no significant synergistic interaction effect between the two modalities (*p* = 0.22). The apoptotic effect level of in vivo cisplatin administration did not differ significantly from the maximum effect observed following a 1-hour in vitro exposure to high cisplatin concentrations. In vivo, the additive effect of cisplatin on IR-induced apoptosis peaked up to 3 days post-administration before returning to baseline by day 6. Consistent with these findings, cisplatin and radiation induced a significant, additive increase in apoptosis within PHA-L-stimulated lymphocytes. Inhibition of ATM has no effect on apoptosis following treatment with cisplatin and IR.

**Conclusions:**

The γ-H2AX pan-nuclear assay provides a valid, reliable method for quantifying radiation- and chemotherapy-induced lymphocyte damage. The time-dependent, additive toxicity observed indicates a critical clinical window (up to 3 days post-cisplatin) for maximum hematologic damage, allowing for optimization of treatment schedules.

**Clinical trial number:**

Not applicable.

## Introduction

Systemic chemotherapy with cisplatin in combination with radiotherapy is one of the most successful radio-chemotherapeutic treatment schedules for tumours [1–4]. The cytotoxic effects of cisplatin are thought to be primarily due to the formation of covalent intra-strand (Pt-GpG and Pt-AG) crosslinks, with a smaller contribution from interstrand crosslinks (G-Pt-G). These adducts modify the DNA structure, thereby inhibiting key cellular processes such as transcription, replication and repair, and can consequently lead to cell death [5].

Cisplatin adducts are usually repaired by either nucleotide excision repair (NER) or homologous recombination (HR) [5, 6]. Consequently, the cytotoxic effect of cisplatin may be influenced by initial or residual adduct levels, i.e. damage formation and repair. A positive correlation has been identified between levels of cisplatin-induced DNA adducts and clinical outcome [7–9]. However, due to the significant variability in adduct levels between individuals [10, 11], it is not always possible to draw definitive conclusions about the relationship between adduct levels and clinical outcomes for specific patients [12, 13].

This variability may be due to discrepancies in repair capacity, cellular signalling, and immunological responses. Therefore, it can be surmised that a cell’s tolerance to residual DNA damage, modulated by these systemic and intracellular networks, determines its fate, leading either to survival or death. Concurrently, the combination of cisplatin-based chemotherapy with radiotherapy represents a highly effective cornerstone in modern oncology, yielding promising therapeutic outcomes in various malignancies, including locally advanced non-small cell lung cancer (NSCLC) [14, 15]. Recent clinical evidence from randomized trials and neoadjuvant protocols continues to demonstrate the superior efficacy of combining platinum compounds with thoracic radiation [14, 15]. However, the success of these intense regimens is frequently limited by severe side effects. Consequently, the identification of reliable biomarkers to monitor individual haematological toxicity remains a critical priority for optimizing patient management in clinical practice.

The conversion of residual cisplatin-induced DNA damage into cytotoxicity is primarily linked to apoptotic cell death [16], which is a genetically regulated process essential for preserving the integrity and homeostasis of multicellular organisms. Apoptosis has been identified as a defence mechanism for eliminating damaged cells. While an increase in the apoptosis of neoplastic cells associated with therapy may improve the outcome, an increase in the apoptosis of non-neoplastic cells, such as lymphocytes, may result in an increased incidence of therapy-related side effects. Nevertheless, the precise mechanism by which pre-treatment with cisplatin alters the sensing and repair of ionising radiation-induced DNA double-strand breaks (DSBs), and the subsequent translation of this damage into cytotoxicity (e.g. apoptotic cell death), remains unclear.

It has been suggested that the non-homologous end joining (NHEJ) pathway may play a role in the radiosensitising effects of cisplatin [17]. The key protein in the NHEJ repair pathway, DNA-PK, has been shown to interact with cisplatin-DNA adducts [18]. Moreover, the degradation of DNA-PK by caspases during apoptosis provides evidence for the involvement of DSB repair in apoptotic signalling [19]. Moreover, recent studies have shown that the DNA damage response is an integral part of the apoptotic pathway [20–24]. One critical factor in signalling from DNA double-strand breaks (DSBs) is the rapid phosphorylation of histone H2AX (γ-H2AX) at or near the lesion site. DSB-dependent phosphorylation of H2AX has been observed following treatment with various exogenous agents, including ionising radiation (IR), topotecan, etoposide, bleomycin and doxorubicin [25–27]. It can also be generated endogenously during programmed DNA rearrangements [28]. γ-H2AX foci are crucial for concentrating repair proteins, such as NBS, Mre11, Rad50, MDC1, 53BP1 and ATM, at DSB-flanking chromatin [29]. We have previously demonstrated that cisplatin interacts with the γ-H2AX signalling pathway, resulting in a significant long-term reduction in radiation-induced γ-H2AX foci formation when lymphocytes are treated in vivo and in vitro [30]. In addition to its role in signalling DSB induction and repair, γ-H2AX has been shown to induce the phosphorylation of the histone variant H2AX as a result of DNA fragmentation during apoptosis [31]. Phosphorylation of H2AX is an early event in apoptosis, occurring concurrently with the activation of caspase-3 [22], the appearance of nucleosomal/oligonucleosomal DNA fragments, as detected by gel electrophoresis, and the externalisation of phosphatidylserine at the outer leaflet of the plasma membrane [31]. A novel form of nuclear γ-H2AX staining, designated the ‘apoptotic γ-H2AX ring’, and intense pan-nuclear staining of the entire nucleus have been observed in apoptotic cancer cell lines [32].

Building on previous research investigating the impact of cisplatin on double-strand break (DSB) repair [30], this study investigates the long-term effects of cisplatin on radiation-induced lymphocyte apoptosis as a marker of hematologic toxicity in cancer patients undergoing standard chemoradiotherapy. Additionally, since lymphocyte proliferation reflects active immune responses during infections, allergies, or autoimmune diseases, we explored how cellular proliferation influences apoptosis. Finally, given that ataxia-telangiectasia mutated (ATM) kinase mediates H2AX phosphorylation during cell death [22], the role of the ATM protein in radiation- and cisplatin-induced lymphocyte apoptosis was also evaluated.

## Materials and methods

### Patients and treatment protocol

This study evaluated 28 cancer patients alongside two healthy control subjects. Approved by the local ethics committee (approval no. 05-2866), all participants provided written informed consent prior to enrollment. The patient cohort presented with tumors located in the intra-thoracic region (n = 21), pelvis (n = 4), and head and neck area (n = 3). All patients underwent concurrent 3D conformal radiotherapy and cisplatin-based chemotherapy (CTx), administered at weekly or longer intervals. Radiation was delivered via a linear accelerator (Varian Inc., Palo Alto, USA) using 6 MV or 15 MV photons, with fractional doses ranging from 1.5 to 2.0 Gy and high dose rates between 1.5 and 4.8 Gy/min. Chemotherapy sessions were scheduled on Mondays, Tuesdays, and occasionally Wednesdays, ensuring no applications occurred on Thursdays; consequently, day 3 featured three radiotherapy fractions following cisplatin delivery. Depending on the regimen, cisplatin doses varied between 20 mg/m² and 50 mg/m², given on days 0 and 7 of a 21–28 day cycle. Specific chemotherapy schedules included.

- Cisplatin monotherapy.
- NSCLC doublet (*n* = 14): Cisplatin (50 mg/m²) and navelbine (15 mg/m²) on days 0 and 7, starting one day after radiotherapy initiation (day − 1).
- SCLC combination (*n* = 4): Cisplatin (45–50 mg/m²) on days 0 and 7, combined with etoposide (100 mg/m²) on days 2, 3, and 4 post-radiotherapy start (day − 1).

At the time of blood sampling, all patients maintained white blood cell counts ≥ 2000/µl, though a complete set of blood samples across all time points was not obtained for every individual.

To isolate the impact of chemotherapy alone on apoptosis, a separate group of six lung cancer patients receiving only systemic treatment was assessed. For these individuals, blood collection occurred at baseline (day − 1), as well as on days 1, 3, and 20 following chemotherapy initiation. Cisplatin was administered at doses between 33 mg and 70 mg per application, delivered four times per week on days 1 to 4 of each weekly treatment cycle. Concomitant medications combined with cisplatin included paclitaxel (275–310 mg; *n* = 3), taxol (261 mg; *n* = 1), alimta (1000 mg; *n* = 1), and etoposide (170 mg; *n* = 1).

Finally, the isolated effect of radiation on apoptotic activity was investigated in five lung cancer patients receiving exclusive radiotherapy. For this subgroup, peripheral blood was drawn at baseline (day − 1) and subsequently on days 1, 3, and 7 during the course of fractionated radiotherapy, which consisted of daily doses ranging from 1.5 to 2.0 Gy.

### Isolation, drug exposure, and in vitro irradiation of lymphocytes

Peripheral blood samples (approximately 5 ml) were drawn from the cubital vein into heparinized tubes, followed by the isolation of mononuclear cells according to previously established protocols [26]. To evaluate in vitro drug exposure and subsequent irradiation, lymphocytes obtained from untreated individuals were pre-incubated for 1 h in supplemented RPMI 1640 medium (containing 10% fetal calf serum and 100 units/ml penicillin/streptomycin; Invitrogen, Carlsbad, USA) with varying cisplatin concentrations (0, 2, 5, 10, 20, and 50 µg/ml; medac, Hamburg, Germany). Proliferation was induced by culturing the cells with 5 µg/ml phytohemagglutinin-L (PHA-L; Sigma, USA) in the same supplemented medium for 72 h.

For experiments involving specific ATM inhibition, lymphocytes from three healthy donors were exposed to 20 µg/ml cisplatin for 1 h. After washing out the drug, the cells were continuously exposed to 50 µM Ku55933 (Selleckchem, Houston, USA) for 24 h, initiating the treatment 1 h prior to a 10 Gy radiation dose. Cell cycle profiles were subsequently evaluated using a Galaxy flow cytometry system (Partec, Germany) following cell fixation in ethanol and DAPI staining.

In vitro irradiation of PBMCs was performed at room temperature (RT) 1 h after cisplatin exposure without replacing the culture medium, utilizing a Co-60 γ-ray source (Philips, Böblingen, Germany) at a dose rate of 1.3 Gy/min. For combined in vivo cisplatin exposure and subsequent in vitro irradiation, blood collections were performed at the specified days post in vivo chemotherapy but invariably prior to that day’s radiotherapy fraction. This timeline guaranteed a minimum interval of 20 h between the preceding in vivo radiation treatment and blood sampling.

### Immunofluorescence analysis and apoptosis quantification

For immunofluorescence staining, cells were fixed at 0.5 h and 4 h post-irradiation according to previously reported methods [26]. The primary antibody used was a mouse anti-γ-H2AX (clone 3F2; Abcam, Cambridge, UK) diluted at 1:400, followed by a goat anti-mouse Alexa 488-conjugated secondary antibody (Invitrogen, Carlsbad, USA) at a 1:500 dilution. Quantitation of γ-H2AX foci was performed by analyzing between 30 and 70 nuclei per blood sample.

To evaluate apoptotic cell death, lymphocyte samples from patients were collected prior to radiation delivery on baseline (day − 1), as well as on days 1, 3, or 6 post-cisplatin administration. Blood collections were timed to ensure that patients had not undergone radiotherapy for at least 20 h, thereby eliminating detectable radiation-induced foci. For comparison, lymphocytes from patients naive to in vivo cisplatin were treated in vitro with cisplatin (2–20 µg/ml) for 1 h, followed by a washout step. Both the in vivo and in vitro drug-exposed lymphocytes were then subjected to radiation doses of 0 Gy, 4 Gy, and 10 Gy.

Following exposure, PBMC samples were maintained in supplemented RPMI 1640 medium for 24 h at 37 °C. Under these culture settings, monocytes adhered to the flask surface within 1 to 2 h, leaving a floating cell fraction consisting primarily of lymphocytes. These non-adherent lymphocytes were harvested, rinsed with PBS, fixed, and labeled with an anti-γ-H2AX antibody for immunofluorescence evaluation. Apoptosis was quantified by determining the percentage of cells displaying pan-nuclear γ-H2AX phosphorylation or characteristic γ-H2AX ring structures [31]. To validate the accuracy of the γ-H2AX apoptotic assay, two additional independent methodological approaches, Caspase-3 assay (BD Biosciences, NJ, USA) and Annexin V assay (ab214485; Abcam, Boston, MA, USA), were performed in strict accordance with the manufacturers’ protocols.

### Data analysis

The effects of cisplatin administration on apoptotic cell death was analysed by analysis of variance. This analysis was done with the general linear model procedure of the SAS software system [33]. A linear model was used to describe the dependence of the average number of radiation induced γ-H2AX foci per nucleus (NmeanH2AX) on radiation dose and the effect of the time of cisplatin application on the slope of the dose - response curve after in vitro irradiation [26]. Blood samples that were taken at day − 3 to day 0 before in vivo cisplatin treatment and had not received cisplatin containing chemotherapy during the last 6 days before the day of blood sampling were classified as d-1. Those taken 1–2 h after cisplatin administration were classified as d0, those taken at days 1–6 after cisplatin administration as d1 - d6 and at day 7 or later for no further cisplatin administration as d ≥ 7. On each day, blood samples were taken just before irradiation. The blood samples were taken around the first cisplatin administration during concurrent radio-chemotherapy and in some patients in addition also around the second administration. In addition, paired t-test or the sign test for pair differences by Dixon and Mood [34] were used to test differences in the measured values between groups after stimulation of lymphocytes.

## Results

### Cisplatin augments radiation-induced lymphocyte apoptosis

**Fig. 1:**
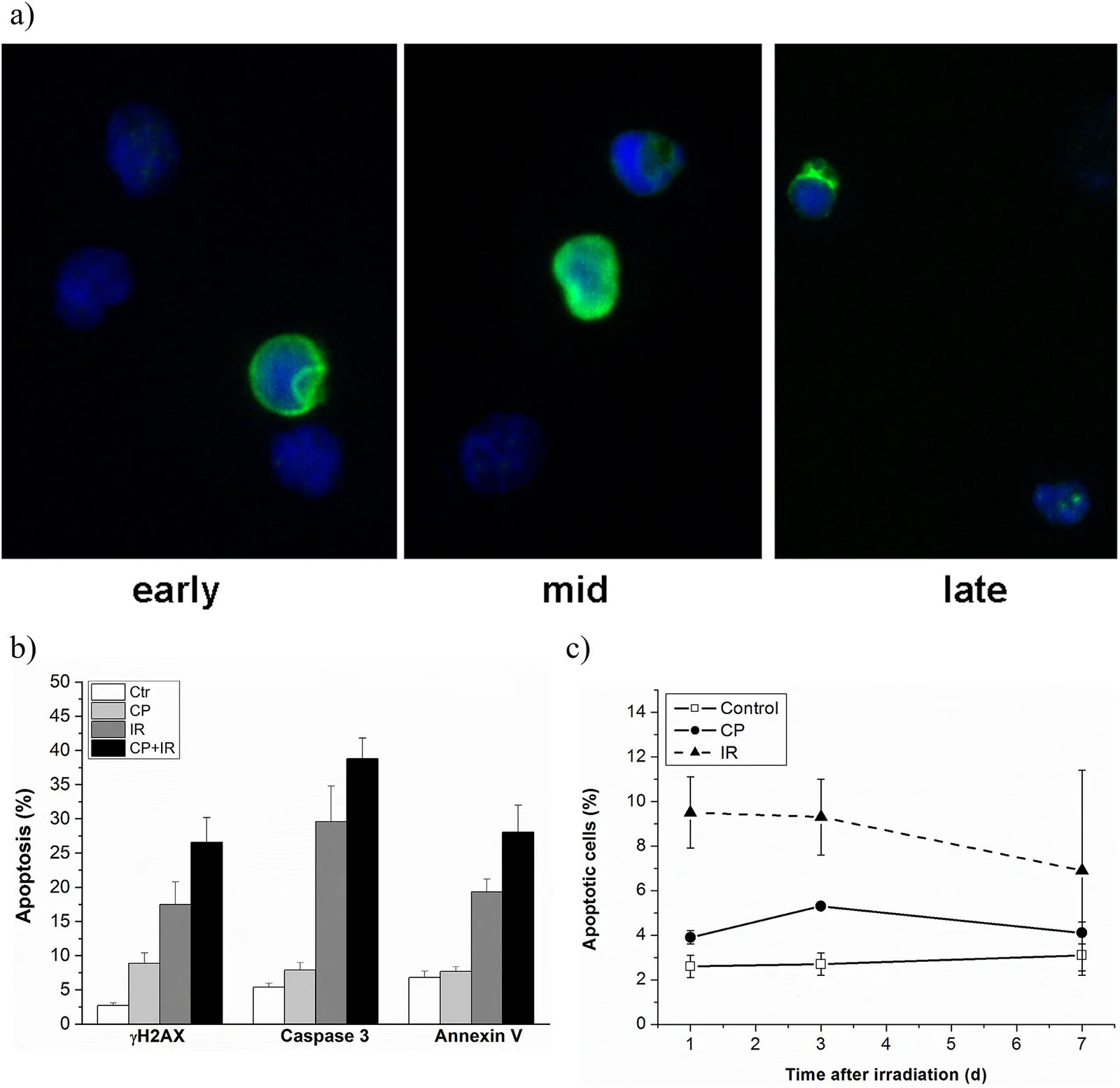
γ-H2AX staining as a surrogate marker for apoptosis. (**a**) Different phases of apoptosis, i.e. early, mid and late, as determined after staining for γ-H2AX. (**b**): Comparison of apoptotic cell death at 24 h after control, 20 µg/ml cisplatin (CP), 10 Gy (IR) and combined treatment (CP + IR), as determined by γ-H2AX staining (*n* = 10), caspase-3 (*n* = 3) and annexin V assay (*n* = 3). (**c**) Time effect on apoptosis as determined by γ-H2AX staining after cisplatin, irradiation and combined treatment schedules

The impact of cisplatin on ionizing radiation (IR)-induced signaling pathways leading to lymphocyte apoptosis was evaluated. Peripheral blood lymphocytes were isolated from healthy individuals and cancer patients who had not received cisplatin within the preceding 6 days. Lymphocytes displaying a peripheral nuclear γ-H2AX ring and intense, pan-nuclear γ-H2AX staining were categorized as apoptotic (Fig. 1a). To validate that the γ-H2AX pan-nuclear assay accurately reflects apoptotic cell death, in vitro-treated lymphocytes from healthy controls were compared against conventional caspase-3 and annexin V assays (Fig. 1b). The γ-H2AX assay data closely mirrored the results of both established assays, showing no statistically significant differences (*p* > 0.05, two-sided **t**-test). Strong Pearson correlation coefficients were observed between γ-H2AX staining and the caspase-3 (*r* = 0.9736) and annexin V (*r* = 0.9780) assays, with corresponding linear regression coefficients of 0.6199 and 1.0047, respectively.

**Fig. 2:**
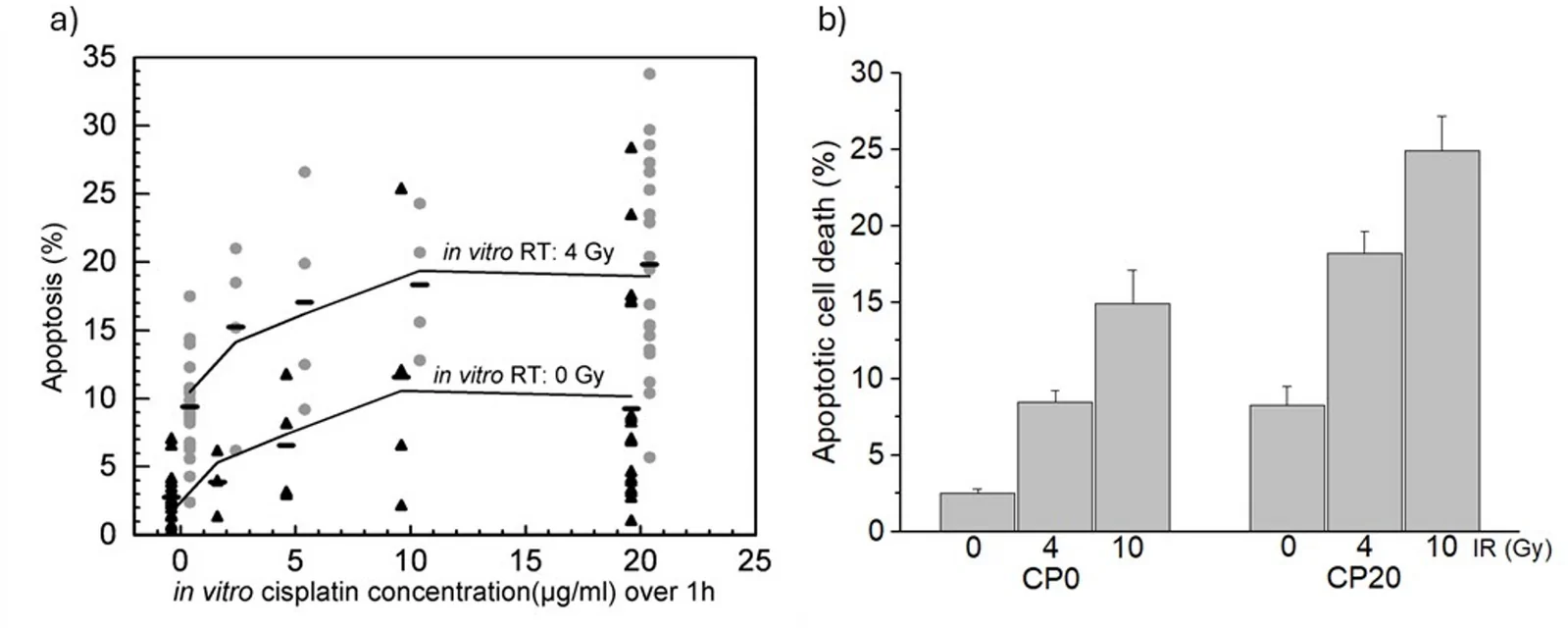
Effect of in vitro cisplatin treatment on radiation induced apoptosis. (**a**) Dose effect relation of in vitro cisplatin exposure in combination with 0 Gy and 4 Gy. Lymphocyte samples from patients not treated with cisplatin or radiotherapy? during the last 6 days and from healthy persons after in vitro cisplatin exposure at the indicated concentration over 1 h and subsequently washed out. Lymphocytes were then irradiated with 0 Gy (filled, black triangels) and 4 Gy (filled, grey circles) and fixed at 24 h after irradiation. (**b**) Dose effect relation of in vitro irradiation in combination with or without cisplatin. The data represent the mean *±* sem of lymphocyte samples from 3–29 patients

Following combination therapy, the percentage of detectable apoptotic cells was higher on day 1 than on days 3 or 7 post-irradiation. This temporal decline likely reflects a loss of γ-H2AX signal integrity alongside advanced cellular disintegration during the late stages of apoptosis (Fig. 1c). Consequently, while the γ-H2AX assay successfully detects apoptotic fractions up to 7 days post-treatment, it provides optimal resolution during early kinetics (< 24 h). In the non-irradiated in vitro treated cisplatin group, the apoptotic fraction increased in a concentration dependent manner at 24 h, reaching an asymptotic plateau of 11.6 *±* 5.0% at 10 µg/ml (Fig. 2a). Combined treatment with cisplatin and radiation increased lymphocyte apoptosis in a strictly additive manner (Fig. 2a, b). Compared to sham-irradiated controls, the in vitro radiation dose exerted a significant main effect on the apoptotic fraction, inducing increases of 9.9 *±* 0.8% and 16.6 *±* 1.5% after 4 Gy and 10 Gy exposures, respectively (*p* < 0.0001, F-test). Both the overall cisplatin concentration effect (*p* < 0.0001, F-test) and the radiation-induced effect (*p* < 0.0001, F-test) were highly significant. Critically, no statistical interaction effect was found between cisplatin exposure and radiation response (*p* = 0.22, F-test), confirming a purely additive toxicity profile.

**Fig. 3:**
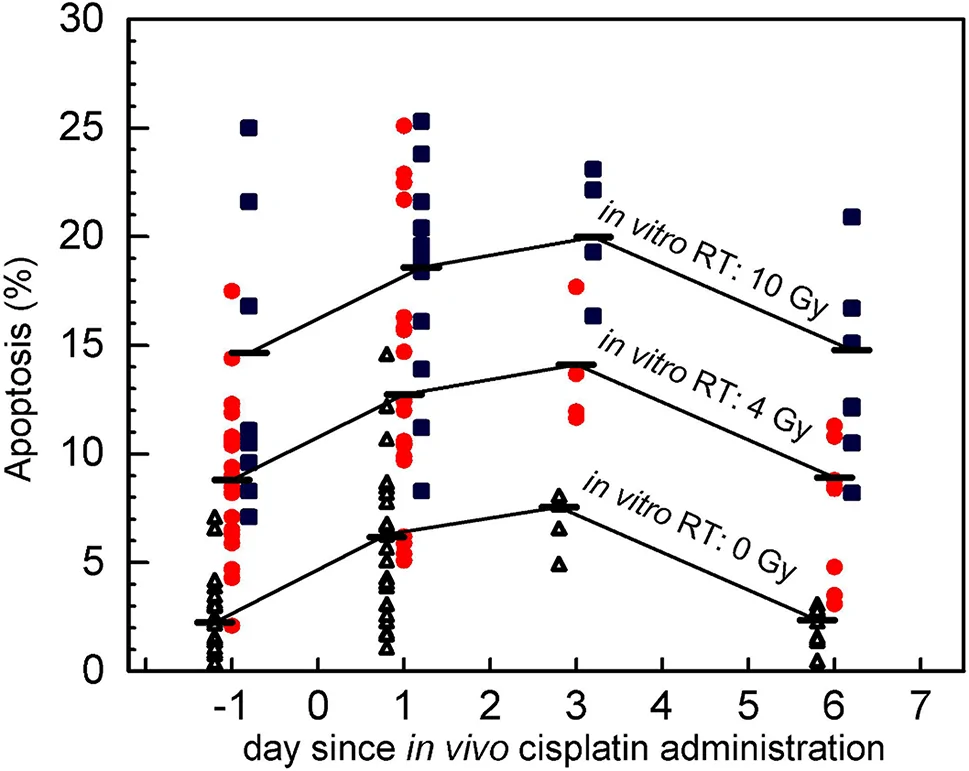
Time dependent effect of in vivo cisplatin exposure and in vitro irradiation apoptosis of lymphocytes. Lymphocyte samples were taken at the indicated time after in vivo cisplatin administration just before the daily radiation dose of the patient. Samples were irradiated in vitro after lymphocyte preparation at the indicated doses of 0 Gy, 4 Gy and 10 Gy and further incubated for 24 h. Black triangles, closed red circles and closed black squares indicate data points from samples irradiated with 0 Gy, 4 Gy, and 10 Gy. Means over all samples of a given treatment group characterized by the same cisplatin and irradiation exposure are indicated by black horizontal bars. The linear regression curves with time since in vivo cisplatin exposure and in vitro radiation dose as covariates connect the mean percentages of apoptosis in the treatment groups estimated by analysis of variance with the classification variables cisplatin exposure condition and radiation dose

Next, the impact of in vivo cisplatin exposure on in vitro radiation-induced apoptosis was assessed in a cohort of nineteen cancer patients. Peripheral blood lymphocytes were sampled at specified time points relative to in vivo cisplatin administration (days − 1, 1, 3, and > 6) and prior to the delivery of the daily clinical radiation fraction. Figure 3 illustrates the apoptotic fractions across these sequential time intervals, both with and without additional in vitro irradiation. A significant overall temporal effect of in vivo cisplatin was observed (*p* < 0.0001, F-test). Compared to baseline (day − 1), the apoptotic fraction increased significantly by 3.4 *±* 1.0% on day 1 and by 4.8 *±* 1.8% on day 3. By day 6 or later, the apoptotic fraction returned toward baseline, showing a non-significant deviation of only 0.5 *±* 1.4% from day − 1.

**Fig. 4:**
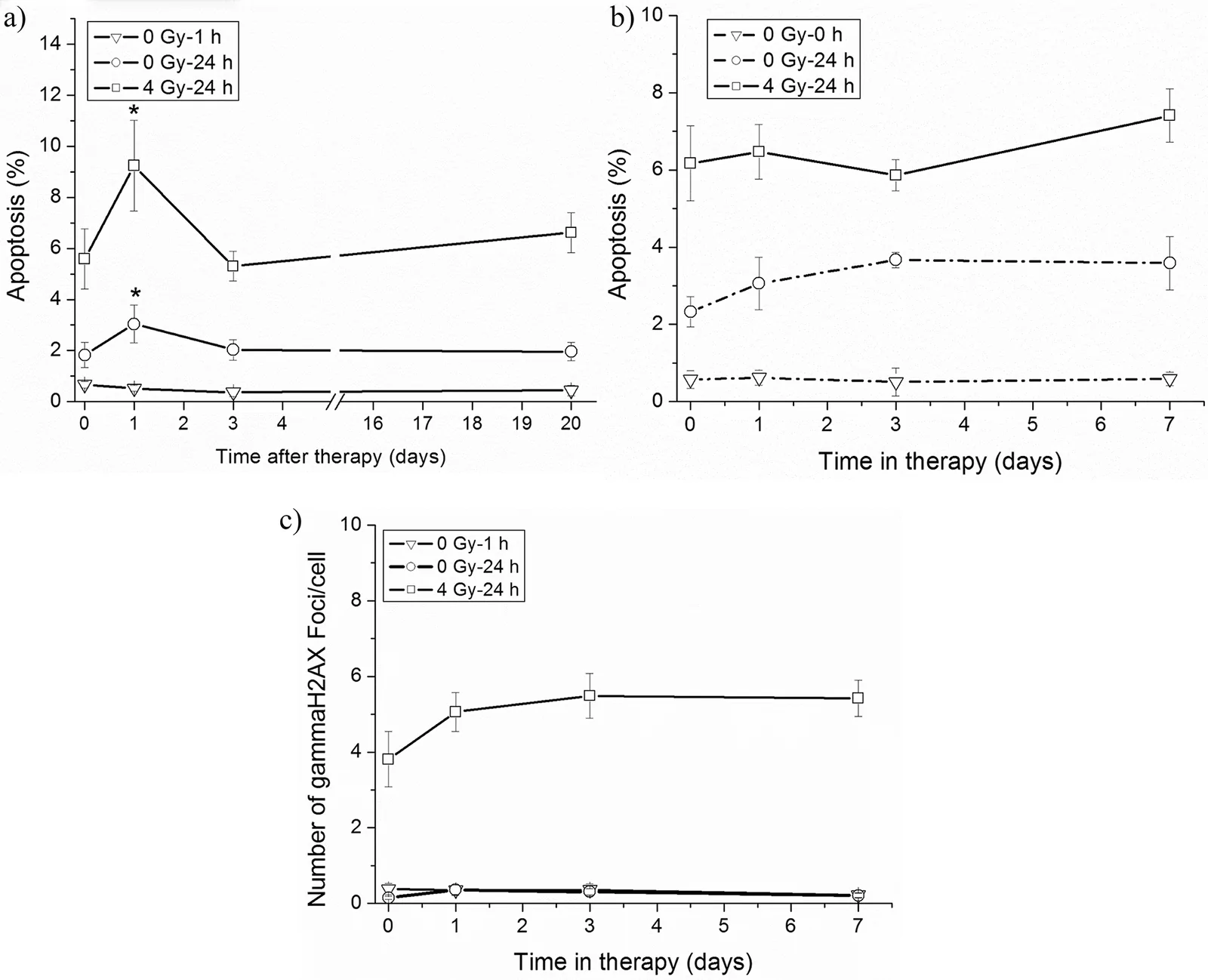
Apoptotic response of ex vivo irradiated lymphocytes from cancer patients. (**a**) The effect of in vivo cisplatin on in vitro radiation induced apoptosis has been measured at 24 h after irradiation with 4 Gy in six NSCLC cancer patients prior to cisplatin treatment at d0, and at d1, d3 and d20 during in vivo cisplatin treatment. (**b**) The effect of in vivo fractionated irradiation on in vitro irradiation induced apoptosis has been measured at 24 h after irradiation with 4 Gy in five NSCLC cancer patients undergoing radiotherapy at d0, and at d1, d7. (**c**) The effect of in vivo fractionated irradiation on the number of γ-H2AX foci at 24 h after ex vivo irradiation with 4 Gy in lymphocytes isolated at d0, and at d1, d7 in five NSCLC cancer patients undergoing radiotherapy. Data represents means *±* sem of the fraction of apoptotic lymphocytes from 6 (**a**) and 5 (**b**) different cancer patients. Significance level at d1 (ranked, sign test) in comparison to the d0 was given with * (< 0.05)

To test the hypothesis that the accumulation of DNA damage-induced apoptosis during the daily fractionated in vivo irradiation of patients can influence the apoptotic response of lymphocytes to in vitro irradiation, control experiments were performed with two selected patient groups. In the 1st group, 6 lung cancer patients treated with cisplatin containing chemotherapy without prior or concomitant radiotherapy were selected. The effect of in vivo cisplatin on in vitro irradiation induced apoptosis has been measured prior to cisplatin treatment at day 0, as well as at day 1 and day 20 after cisplatin treatment (Fig. 4a). Again, a significant increase in the fraction of apoptotic cells with + 2.35 *±* 0,87% (*p* < 0.01019) at 24 h after irradiation was evident at day 1 but not at day 20 after cisplatin treatment.

A second group of five cancer patients undergoing radiotherapy without cisplatin was selected to explore whether apoptotic cells accumulate during daily in vivo fractionated irradiation at an integral body dose ranging from 0.008 to 1.286 Gy, which could influence the apoptotic response of lymphocytes to in vitro irradiation. As can be seen in Fig. 4b, there was no significant effect of daily in vivo irradiation on the fraction of apoptotic cells, measured at 24 h after in vitro irradiation of lymphocytes for 1d or longer before taking of the blood samples. In order to estimate the effect of daily irradiation on the number of remaining DNA double-strand breaks at 24 h after in vitro irradiation with 4 Gy, the number of γ-H2AX foci was determined in the same slides after staining with an anti-γ-H2AX antibody. As demonstrated in Fig. 4c, the data evidently show a minor increase in the number of residual gH2AX foci on day 1 following the commencement of radiotherapy. However, no further increase was observed thereafter during the course of the experiments.

Overall, these data show that in vivo cisplatin treatment induces long-term effects in peripheral blood lymphocytes that remain in the circulation for prolonged times of *≥* 1 days. These long-term effects are additive to radiation induced apoptosis after subsequent in vitro irradiation and 24 h of in vitro incubation. After in vivo irradiation at mean whole body doses of < 1.3 Gy per fraction, no accumulation of lymphocytes that are more sensitive to apoptosis after incubation for 24 h with or without in vitro irradiation was detected.

### Cisplatin additively increases radiation-induced apoptosis in proliferating lymphocytes

**Fig. 5:**
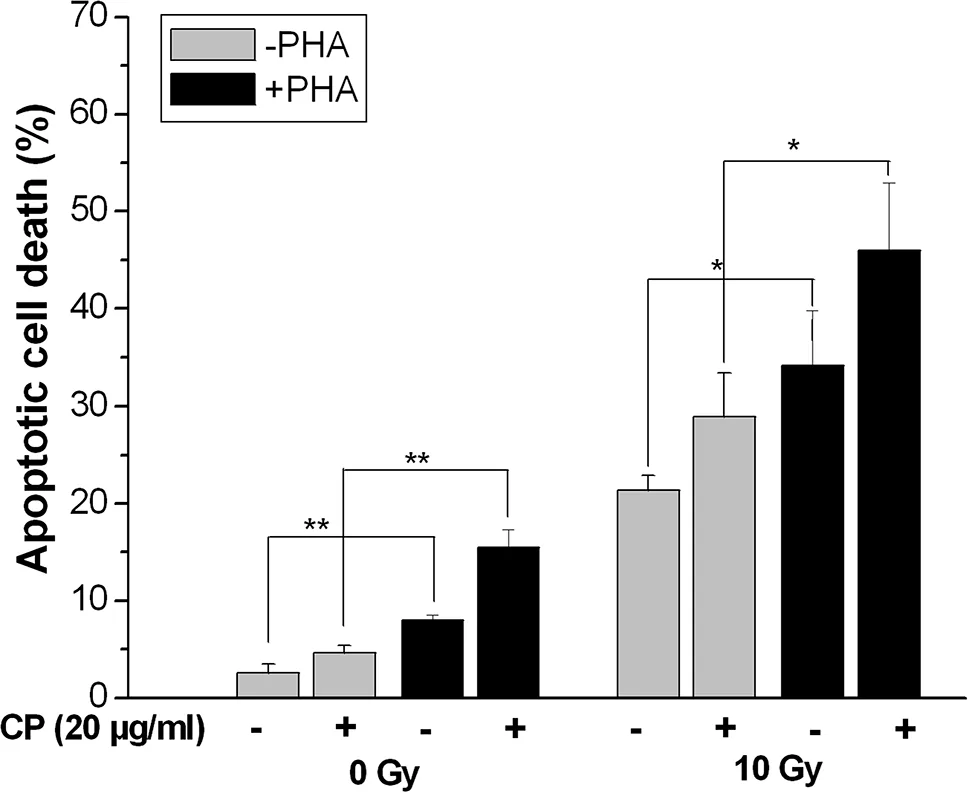
Effect of cisplatin and radiation exposure on apoptosis of proliferating lymphocytes. Samples from patients not treated with cisplatin during the last 6 days and from healthy persons were treated for 72 h with or without 5 µg/ml PHA-L in order to stimulate proliferation. At the end of treatment, lymphocytes were treated with 20 µg/ml cisplatin over 1 h and subsequently washed out and irradiated with 0–10 Gy. Apoptotic fractions of lymphocytes at the end of a 24 h in vitro incubation period were determined. The data represent the mean *±* sem of 7 independent lymphocyte samples from 5 cancer patients. Significance levels of the stimulated samples (ranked, sign test) in comparison to the respective non-stimulated samples are given with * (< 0.05), ** (< 0.01)

To evaluate whether cellular proliferation influences the interaction between cisplatin and ionizing radiation (IR), lymphocytes were treated with phytohemagglutinin-L (PHA-L) to stimulate mitogenesis. The proliferating cell fraction quantified by the percentage of cells in S- and G2-phases increased from 9 ± 5% to 44 ± 5% at 72 h after stimulation. Stimulated and unstimulated lymphocytes from 5 cancer patients were in vitro treated with 20 µg/ml cisplatin 1 h before irradiation with 10 Gy and apoptotic cell death was determined 24 h thereafter (Fig. 5). PHA-L stimulation significantly increased background apoptosis from 2.6 + 0.9% in untreated resting cells to 8.0 + 0.5% in proliferating cells (*p* < 0.01, paired t-test). Proliferative activity similarly elevated apoptosis rates following single-modality exposures, increasing cisplatin-induced cell death from 4.6 + 0.8% to 15.5 + 1.8% (*p* < 0.01, paired t-test) and radiation-induced cell death from 21.4 + 1.5% to 34.1 + 5.7% (*p* < 0.05, paired t-test). Furthermore, the combined treatment cohort exhibited a significant proliferation-dependent increase in apoptosis, rising from 28.9 + 4.5% to 46.0 + 6.9% (*p* < 0.01, paired t-test). Mechanistically, treating proliferating lymphocytes with 20 µg/ml cisplatin significantly suppressed homologous recombination repair, reducing the number of radiation-induced Rad51 foci at 4 h post-10 Gy exposure from 5.0 + 0.7 to 2.1 + 0.8 per cell (*p* < 0.05, paired t-test).

In conclusion, mitogenic stimulation with PHA-L increases the baseline apoptotic susceptibility of lymphocytes across all cohorts, rendering the absolute cell death rates higher. Crucially, because this increase occurs symmetrically across mono- and combination therapies, the fundamentally additive toxic relationship between cisplatin and radiation remains entirely independent of lymphocyte proliferation status.

### Inhibition of ATM signaling does not affect lymphocyte apoptosis

**Fig. 6:**
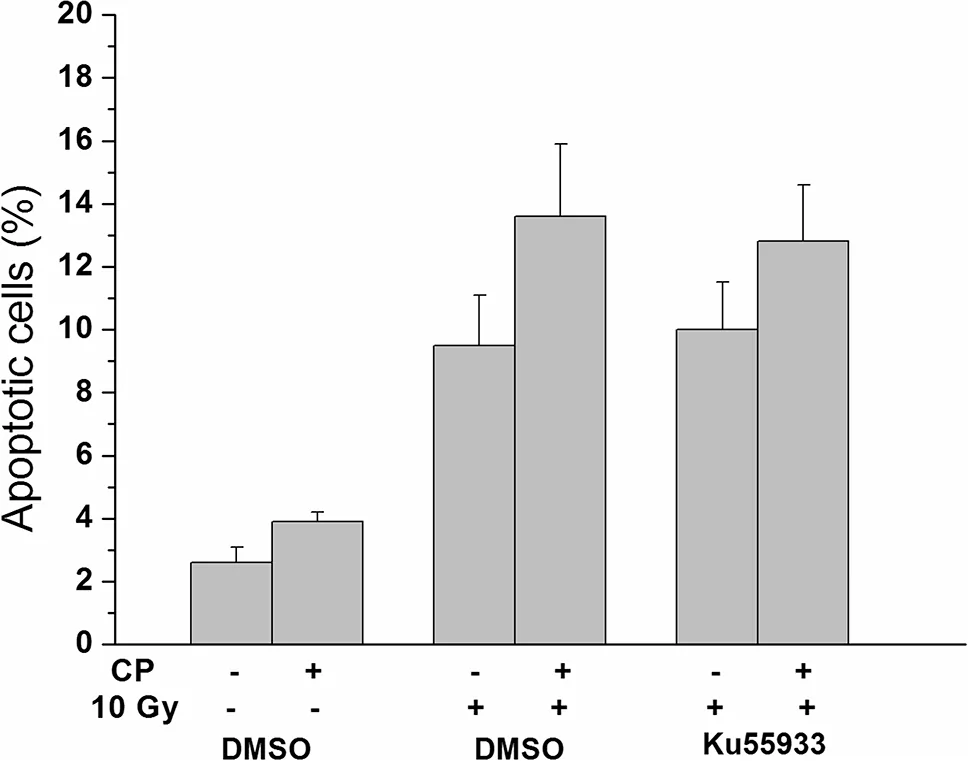
Effect of *ATM* on radiation and cisplatin induced apoptosis in lymphocytes. Lymphocyte probes were collected from 3 healthy individuals and were in vitro treated for 1 h with 0 µg/ml (CP0) or 20 µg/ml cisplatin (CP20), washed out and treated with Ku55933 (50 µM). At 1 h later cells were irradiated with 10 Gy and lymphocytes with excess γ-H2AX signal as a measure for apoptosis was determined 24 h later. Means + sem of 5 experiments are shown

To evaluate whether ataxia-telangiectasia mutated (ATM) signaling pathways are involved in driving the excessive H2AX phosphorylation that characterizes apoptotic cell death, the specific small-molecule ATM inhibitor KU-55,933 was utilized. Lymphocytes isolated from three healthy individuals were treated in vitro for 1 h with 20 µg/ml cisplatin, washed, and then continuously exposed to 50 µM KU-55,933 for 24 h, beginning 1 h prior to a 10 Gy radiation fraction. Consistent with the previous cohorts, cisplatin additively increased radiation-induced apoptosis (Fig. 6). However, pre-treatment of resting lymphocytes with KU-55,933 before irradiation exerted no significant effect on the apoptotic fraction following either single-modality ionizing radiation or combined treatment protocols (*p* > 0.05). Consequently, these data indicate that ATM signaling is not required for the excessive, pan-nuclear phosphorylation of H2AX during apoptosis in peripheral lymphocytes.

## Discussion

In the clinical setting, cisplatin is frequently combined with ionizing radiation (IR) [1–4]. However, the precise interaction between these two modalities, particularly how it differs between proliferating and resting cells, remains to be fully established. Cisplatin-induced crosslinks, when resolved, trigger replication stress and force cells into apoptosis [35]. While cisplatin is known to cause chromosomal damage following both in vitro [36] and in vivo [37] exposure, the combined impact of cisplatin and IR on peripheral blood mononuclear cells (PBMCs) is not yet completely understood. PBMCs comprise immune cells, predominantly monocytes and lymphocytes (including T, B, and NK cells), that typically reside in a resting state. In human PBMCs, cisplatin has been shown to reduce the formation of radiation-induced γ-H2AX repair foci, decrease micronuclei formation, and increase apoptotic cell death [38, 39].

In the present study, we evaluated the consequences of in vivo and in vitro cisplatin exposure, combined with in vivo and in vitro irradiation, on the apoptotic death of PBMCs isolated from cancer patients. We observed a dose-dependent increase in apoptosis up to 10 µg/mL of in vitro cisplatin exposure, which plateaued at higher concentrations. Similarly, ionizing radiation induced a dose-dependent increase in apoptosis up to 10 Gy, aligning with previous reports on radiation-induced lymphocyte apoptosis [38, 40, 41]. Notably, the apoptotic effect level of in vivo cisplatin administration did not differ significantly from the maximum effect observed following a 1-hour in vitro exposure to high cisplatin concentrations. It is important to note that the pan-nuclear γ-H2AX assay encompasses distinct morphological stages of cell death, shifting from early peripheral ring-like staining to late-stage complete pan-nuclear coverage. While the early ring formation is highly indicative of initiated apoptosis, the late-stage global pan-staining reflects massive, widespread DNA fragmentation. At this advanced phase of cellular degradation, the assay does not permit a clear distinction between late apoptosis and necrosis, as both pathways result in total nuclear H2AX phosphorylation. Consequently, the quantified pan-nuclear fraction represents a cumulative readout of total advanced cytotoxicity induced by the combined cisplatin and radiation treatment.

Following combined treatment schedules, in vivo or in vitro cisplatin exposure paired with in vitro irradiation of PBMCs yielded only an additive effect on apoptosis. This additive effect of IR and cisplatin on lymphocyte apoptosis aligns with findings by Zahnreich et al. [38]. However, the cell cycle phase at the time of irradiation can markedly influence both apoptotic cell death [42] and the activation of Rad51-dependent homologous recombination [43]. Consequently, the cisplatin-mediated reduction of homologous recombination, driven by decreased Rad51 foci formation [44], may represent a mechanism by which cisplatin selectively radiosensitizes proliferating cells. Supporting this hypothesis, a synergistic effect of cisplatin and radiation on cell death has been observed in several proliferating tumor cell lines [45, 46]. Lymphocyte proliferation, which occurs during active immune responses (e.g., infections, allergies, or autoimmune diseases), is a critical factor when considering the interactive effects of cisplatin and IR on patient immunity. Among lymphocyte subpopulations, CD8 + T-lymphocytes (cytotoxic cells) are highly predictive of radiation-induced late toxicity [47–49] and patient survival [49, 50]. Therefore, evaluating whether apoptotic cell death is differentially regulated in specific lymphocyte subsets following combined cisplatin and IR treatment is of particular interest. Although investigating these subpopulations was outside the scope of the present study, it will be comprehensively evaluated in a forthcoming investigation.

Consequently, clarifying the role of cisplatin in modulating these immunological responses remains of paramount importance. While we focused on apoptotic nuclear DNA fragmentation via γ-H2AX induction, the resulting nuclear and mitochondrial DNA fragments can act as damage-associated molecular patterns (DAMPs) that drive downstream inflammatory and innate immune cascades in vivo. The physiological impact of this oxidative and organelle insult is underscored by recent strategies showing that activating cytoprotective networks, such as the Nrf2 pathway by antioxidants like hesperidin, can prevent cisplatin-induced tissue damage [51]. Although direct in vivo tracking of mitochondrial dynamics and immune networks was not performed here, our findings establish a necessary baseline. Future studies focus on how the interplay between lymphocyte DNA fragmentation and mitochondrial signaling shapes the individual immunological landscape during radiochemotherapy.

The findings of the present study demonstrate that the apoptotic fraction increases in proliferating compared to non-proliferating lymphocytes, consistent with previous observations by Vilasova et al. [47]. Cisplatin further enhanced the fraction of radiation-induced apoptosis. However, the data demonstrated a strictly additive effect, indicating a lack of synergistic interaction between cisplatin and IR on the apoptotic endpoint at 24 h post-treatment. These findings regarding the impact of cisplatin and radiation on lymphocyte apoptosis align with clinical observations, which indicate a moderate escalation in toxicity following concurrent radiochemotherapy compared to either modality administered alone [52, 53]. In addition, cisplatin attenuates Rad51 foci formation, which serves as a marker for homologous recombination in proliferating (i.e., PHA-L-stimulated) lymphocytes. As a novel mechanism of interaction, we previously demonstrated a significant decrease in the number of radiation-induced γ-H2AX foci in lymphocytes pre-treated with cisplatin. The kinetic profiles for the effects of in vivo cisplatin on γ-H2AX foci formation (a marker for DNA repair signaling) and pan-nuclear γ-H2AX staining (a marker for apoptotic cell death) are inversely related. For both endpoints, the maximum effect was observed at day 1, followed by a decline to baseline levels by day 6 or later. These data demonstrate that the reduced formation of ionizing radiation-induced γ-H2AX foci following cisplatin exposure reflects impaired repair signaling efficacy. This reduction is predictive, at the very least, of subsequent apoptotic cell death in PBMCs.

Regarding kinetic profiles, the data show that cisplatin-sensitized peripheral blood mononuclear cells (PBMCs) persist in the circulation for up to three days post-treatment. Consequently, the impact of in vivo cisplatin exposure on apoptosis following in vitro irradiation can be reliably detected within this timeframe. Furthermore, the kinetics of in vitro radiation-induced apoptosis in lymphocytes yield comparable results regardless of whether patients undergo high-dose in vivo cisplatin monotherapy or concurrent radiochemotherapy. Notably, the data demonstrate that fractionated daily in vivo radiotherapy at mean whole-body doses of < 1.3 Gy per fraction does not sensitize lymphocytes to apoptosis upon subsequent in vitro irradiation. This lack of sensitization is presumably because the dose per fraction is insufficient to trigger apoptotic pathways, or because the fraction of apoptotic cells remains below the detection limit. Our findings also reveal that daily fractionated in vivo irradiation up to day 7 does not significantly alter the number of unrepaired double-strand breaks (DSBs) at 24 h following a 4 Gy in vitro irradiation challenge. The pan-nuclear g-H2AX assay for apoptotic cell death is less sensitive than the g-H2AX foci assay for DNA damage repair signaling regarding radiation dose estimation. While the γ-H2AX apoptosis assay is unsuitable as a biodosimeter for in vivo radiation, it is sufficiently sensitive to quantify the modulating effects of in vivo or in vitro cisplatin exposure on radiation-induced apoptosis. Most importantly, this approach enables the simultaneous measurement of repair signaling (γ-H2AX foci) and apoptosis (γ-H2AX pan-nuclear staining) within the same sample, utilizing an identical immunofluorescence staining and preparation protocol.

Regarding the molecular signaling of apoptosis, cisplatin-mediated phosphorylation and stabilization of p53, and the subsequent activation of the pro-apoptotic protein BAX [55], most likely explain the pro-apoptotic effects of cisplatin in cancer cells. While p53 activation can involve ATM signaling [54, 55], we previously demonstrated that cisplatin does not affect radiation-induced ATM activity in PBMCs [30]. Consistently, the results of the present study show that pre-treatment of PBMCs with the specific ATM inhibitor Ku55933 has no significant impact on apoptosis, as measured by pan-nuclear γ-H2AX foci staining.

While this finding might initially contrast with standard models in proliferating tumor cell lines [56, 57], it aligns with specific characteristics of resting (G_0_) peripheral lymphocytes and the nature of the apoptotic g-H2AX assay [58]. This absolute dependence on cell-cycle distribution has been heavily reinforced by recent findings from Qiu et al. [59], who demonstrated that while cisplatin-DNA adducts accumulate uniformly across all cell cycle phases, the generation of DSBs is strictly restricted to the S-phase as a result of replication fork stall and collapse. This mechanistic insight directly underpins our observations in primary human PBMCs, which naturally reside in a non-replicating G0 state. Because these resting lymphocytes lack the replication-dependent machinery required to translate platinum adducts into severe S-phase-specific DSB evolution, they display a distinct toxicological profile that resists classic cell-cycle-targeted radiosensitization cascades [55].

In those cycling tumor environments, ATM is strictly required to orchestrate cell cycle checkpoints and repair active double-strand breaks before replication; blocking this pathway forces cells into mitotic catastrophe and subsequent cell death [56, 57]. However, because primary peripheral lymphocytes do not undergo active cell division or face mitotic checkpoints, their apoptotic trajectory following combined treatment is largely decoupled from upstream ATM-mediated repair checkpoints. This highlights a critical therapeutic window where ATM inhibition selectively radiosensitizes rapidly dividing tumor tissues while leaving non-proliferating healthy mononuclear cells largely unaffected. Although ATM is crucial for early localized γ-H2AX foci formation flanking DNA double-strand breaks during active repair, alternative PI3K-like kinases, most notably DNA-PK, are primarily responsible for driving the massive, global pan-nuclear phosphorylation of H2AX during the execution phase of apoptotic DNA fragmentation [20, 29]. Indeed, ATM activity has been shown to be dispensable for apoptotic γ-H2AX pan-nuclear staining in human cells following apoptotic triggers [21]. In addition, non-proliferating lymphocytes are highly sensitive to transcriptional stress and alternative caspase activation pathways. Persistent cisplatin crosslinks can block RNA polymerase progression, triggering transcription-coupled signaling that leads to p53-independent or ATM-independent apoptosis [5, 16]. Consequently, alternative pathways compromised by cisplatin exposure, such as nucleotide excision repair, rather than classic upstream ATM checkpoints, likely dictate the apoptotic fate in these resting primary cells [6].

Beyond traditional nuclear DNA damage, the cytotoxic efficacy of cisplatin is heavily dictated by non-nuclear mechanisms, most notably mitochondrial dysfunction. As highlighted recently, reactive oxygen species (ROS) generation represents a central pathway through which cisplatin exerts its toxicity [60] with mitochondria being a uniquely vulnerable to this oxidative insult. This mitochondrial collapse strongly cooperates with DNA damage repair deficiencies to accelerate the activation of downstream apoptotic cascades in primary cells. This critical involvement of mitochondrial insult in overall cisplatin cytotoxicity is historically well-documented across various experimental models [5, 61]. Crucially, this drug-induced mitochondrial stress does not act in isolation; it plays an instructive role in shaping innate and adaptive immune responses. Mitochondrial damage leads to the release of damage-associated molecular patterns (DAMPs) and modulates cellular immunogenicity, which is vital for the synergistic clinical outcomes of cisplatin when combined with radiotherapy. This is consistent with recent clinical data demonstrating that cisplatin-based regimens induce profound immunomodulatory changes that significantly improve patient survival during combination therapies [62]. Beyond its immunomodulatory role, cisplatin-compromised mitochondria generate an initial wave of excessive reactive oxygen species (ROS), which can trigger a self-amplifying cascade known as ‘ROS-induced ROS release’ accelerating oxidative injury [63]. The therapeutic relevance of this pathway is underscored by recent strategies showing that preserving mitochondrial function or utilizing mitochondrially-targeted antioxidants can successfully mitigate cisplatin-induced tissue injuries [64]. Integrating these multi-layered mitochondrial and nuclear injuries provides a more comprehensive view of how primary cells respond to cisplatin combined with ionizing radiation.

In conclusion, our study demonstrates that the combined effect of cisplatin and ionizing radiation on peripheral blood lymphocytes is strictly additive. Crucially, this additive toxicity is entirely independent of the proliferation status of the lymphocytes, manifesting consistently in both resting G_0_ and proliferating cells. Furthermore, the pan-nuclear γ-H2AX assay proved to be a reliable and advantageous marker for quantifying advanced cytotoxicity, which operates independently of classic upstream ATM signaling checkpoints.

## Data Availability

The datasets used and/or analyzed during the current study are available from the corresponding author on reasonable request.
